# Dataset smartphone usage of international tourist behavior

**DOI:** 10.1016/j.dib.2019.104610

**Published:** 2019-10-05

**Authors:** Jack Febrian Rusdi, Sazilah Salam, Nur Azman Abu, Budi Sunaryo, Rohmat Taufiq, Lita Sari Muchlis, Trisya septiana, Khairil Hamdi, Arianto Arianto, Benie Ilman, Desfitriady Desfitriady, Frans Richard Kodong, Anik Vega Vitianingsih

**Affiliations:** a Department of Informatics Engineering, Sekolah Tinggi Teknologi Bandung, Bandung, 40235, Indonesia; b Faculty of Information and Communication Technology, Universiti Teknikal Malaysia Melaka, Melacca, 76100, Malaysia; c Department of Electrical Engineering, Engineering Faculty, Andalas University, Limau Manis, Padang, 25163, Indonesia; d Department of Informatics Engineering, Engineering Faculty, University Muhammadiyah of Tangerang, Tangerang, 15117, Indonesia; e Department of Informatics Management, IAIN Batusangkar, Tanah Datar, 27213, Indonesia; f Department of Informatics Engineering, Engineering Faculty, Lampung University, Bandar Lampung, 35145, Indonesia; g Department of Information System, STMIK Jayanusa, Padang, 25112, Indonesia; h Department of Informatics Engineering, Universitas Langlangbuana, Bandung, 40261, Indonesia; i Economic Faculty, Universitas Nasional PASIM, Bandung, 40175, Indonesia; j Program Studi Teknik Informatika, UPN “Veteran” Yogyakarta, Yogyakarta, 55281, Indonesia; k Department of Informatics, Universitas Dr.Soetomo, Surabaya, 60118, Indonesia

**Keywords:** Information and communication technology, Smart tourism, Smartphone, Mobile computing, Tourist behavior

## Abstract

This article contains dataset on the behavior of international tourists when traveling is related to 1) tourist demographics, 2) things that affect tourists to choose travel destinations when planning, 3) use of mobile data while traveling, 4) how to get internet access while traveling, 5) social media used during traveling, and 6) behavior of smartphone use for tourists during traveling. The raw data presented here can be used as material to analyze the behavior of international tourists related to any media that affects international tourists in planning their trips, and how they behave during traveling. This data is a source of raw data from our research on smartphones and international tourist behavior, besides being used for various other research purposes.

**Table:** Specifications Table

**Subject**	Computer Network and Communications
Specific subject area	ICT in Tourism
Type of data	Text in Data Sheet, Questionnaire Form, Tourist Response.
How data were acquired	The survey, analytics, self-report questionnaires.
Data format	Raw data in datasheet format, Excel compatible
Parameters for data collection	Demographic Behavior for international tourist while traveling
Description of data collection	Raw data collection through a questionnaire about the behavior of international tourists on smartphone use.
Data source location	Bandung, Indonesia.
Data accessibility	Repository name: Mandeley Data Data identification number: https://doi.org/10.17632/zwzb8hzc9j.1 Direct URL to data: https://data.mendeley.com/datasets/zwzb8hzc9j/1

**Table:** 

**Value of the Data** • This dataset is useful for those who want to acquire an international tourist behavior. • This dataset can provide benefits for ICT developers as well as Tourism Stakeholder. • This dataset is easy to process for further information. • Available data provide the behavior of international tourists on technology usage.

## 1 Data

**Fig. 1:**
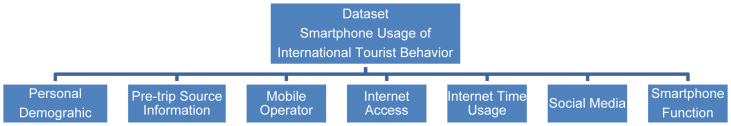
Data group in the dataset.

The dataset is the result of the distribution of response from international tourists related to the ICT— a source of input to infer tourist behavior used this dataset. The data are mainly related to the use of information and communication technology [[1], [2], [3], [4], [5], [6], [7]]. The dataset consists of seven groups, as shown in Fig. 1.

**Fig. 2:**
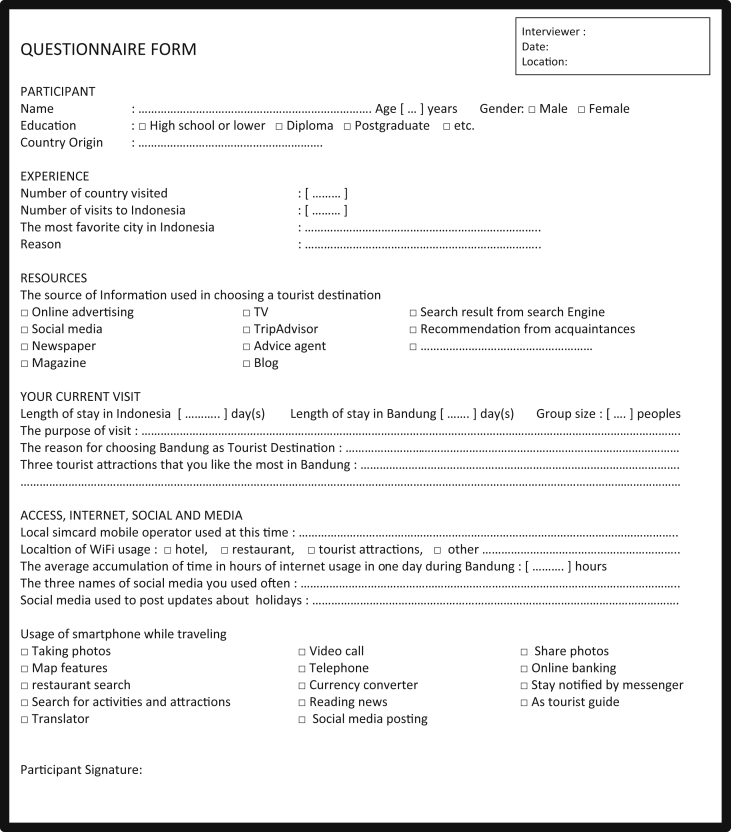
Questionnaire form.

Each group stores specific data fields within the groups. The criteria for each behavioral group stored in each record. Following Table 1, Table 2, Table 3, Table 4, Table 5, Table 6, Table 7 in the Questionnaire form (Fig. 2) as an essence of fields used in storing the results of the questionnaire is acquired one by one.

**Table 1:** Group fields of Tourist Personal Demographic.

Field Name	Type	Description
p_age	Number	Age of tourist
p_gender	Options “M” or “F”	Gender of tourist
p_edu	Options “L”, “D”, “PG” or “ETC”	The education level of tourist. The contents are in the form of choices, L: under the university, D: Diploma, PG: Postgraduate, ETC: other options.
p_country	Text	Country origin of tourist

**Table 2:** Group fields of Tourist Pre-trip Source Information.

Field Name	Type	Description
pre_OA	Boolean	Online Advertising,
pre_SM	Boolean	Social Media
pre_NP	Boolean	Newspaper
pre_Mg	Boolean	Magazine
pre_TV	Boolean	Television
pre_TA	Boolean	Tripadvisor
pre_AA	Boolean	Advice Agent
pre_Bl	Boolean	Blog
pre_SE	Boolean	Search Engine
pre_rec	Boolean	Recommendation
pre_desc	Boolean	Description additional information

**Table 3:** Group fields of Tourist Mobile Operator while traveling.

Field Name	Type	Description
m_local	Boolean	Local operator usage by tourist
m_roaming	Boolean	Activates roaming facilities from the operator of the country of origin

**Table 4:** Fields in the group of Tourist Internet Usage Location.

Field Name	Type	Description
internet_hotel	Boolean	Internet usage at the hotel by tourist
Internet_restaurant	Boolean	Internet usage at restaurant by tourist
Internet_tourist_attraction	Boolean	Internet usage at a tourist attraction by tourist

**Table 5:** Group fields of Tourist Internet-Connected and Usage Time While Traveling.

Field Name	Type	Description
Internet_daily_usage	Number	Total hours of daily usage and connected to the internet while traveling by tourist

**Table 6:** Group fields of Tourist usage of Social Media While Traveling.

Field Name	Type	Description
Soc_med	Text	Tourist social media name used while traveling

**Table 7:** Group fields of Tourist Smartphone Function While Traveling.

Field Name	Type	Description
sp_TP	Boolean	Taking photos
sp_MF	Boolean	Map features
sp_RS	Boolean	Restaurant search
sp_SAA	Boolean	Search of attraction
sp_Tr	Boolean	Translator
sp_VC	Boolean	Video Call
sp_Tl	Boolean	Telephone
sp_CC	Boolean	Currency converter
sp_SMP	Boolean	Social media posting
sp_RN	Boolean	Reading news
sp_SP	Boolean	Share photos
sp_OB	Boolean	Online banking
sp_WA	Boolean	Whatsapp
sp_ATG	Boolean	As tour guide

Each group present of each topic. Personal data stored in a table based on these groups:

1. Personal demographic. The demographics of each tourist participating in completing the questionnaire (see Table 1)
2. Pre-trip source information. Pre-trip source information. Information used by tourists when planning a trip before actual (see Table 2)
3. Mobile operator. The use of mobile operators by tourists to connect to the internet during the trip takes place (see Table 3)
4. Internet access. The source of internet access during a trip (See Table 4)
5. Internet usage time. Group fields of Tourist Internet-Connection and usage duration while traveling (See Table 5)
6. Social media. Social media used by tourists during a trip (See Table 6)
7. Smartphone Function. Selection of services from a smartphone by a tourist on a trip (See Table 7)

## 2 Experimental design, materials, and methods

Application programs that can be used to open, process, and display the query datasets are compatible with Microsoft programs that can open data in XLSX format. To use this data, the user can retrieve it from the dataset stored on Mendeley's repository [8].

Data material was obtained from the results of the distribution of questionnaires on foreigners and tourists in Bandung [9,10] in the period 11 April 2019 to 28 May 2019.

To process and experiment with data, including doing it by filtering, sorting by using the general formula that is in the data processor.

Based on existing data, data processors can find some results according to the wishes included in the dataset, for example, particular country tourist behavior, age-based behavior, gender-based behavior, or behavior based on education level. Processing is also combined based on several other categories.

Thus, the data contained in this dataset can be an input for various parties related to the behavior of international tourists to be able to travel [11]. This data is useful for a variety of research carried out in the field of ICT [12], especially in the area of tourism research.

## Transparency document

As a form of transparency related to this article, data can be found online, namely through the repository provided by Mendeley with the address https://doi.org/10.17632/zwzb8hzc9j.1.
