# Supplementary material for: Dataset smartphone usage of international tourist behavior
Source: Data Brief. 2019 Oct 5;27:104610. doi: 10.1016/j.dib.2019.104610 (PMC6806464; doi:10.1016/j.dib.2019.104610)
Supplement: Multimedia component 1 [file mmc1.docx]

QUESTIONNAIRE FORM

Interviewer :

Date:

Locatonon:

PARTICIPANT

Name : ………………………………………………………………. Age [ … ] years Gender: □ Male □ Female

Education : □ High school or lower □ Diploma □ Postgraduate □ etc.

Country Origin : ………………………………………………….

EXPERIENCE

Number of country visited : [ ……… ]

Number of visits to Indonesia : [ ……… ]

The most favorite city in Indonesia : ………………………………………………………………..

Reason : ………………………………………………………………..

RESOURCES

The source of Information used in choosing a tourist destination

□ Online advertising □ TV □ Search result from search Engine

□ Social media □ TripAdvisor □ Recommendation from acquaintances

□ Newspaper □ Advice agent □ ………………………………………………

□ Magazine □ Blog

YOUR CURRENT VISIT

Length of stay in Indonesia [ ……….. ] day(s) Length of stay in Bandung [ ……. ] day(s) Group size : [ …. ] peoples

The purpose of visit : …………………………………………………………………………………………………………………………………………………….

The reason for choosing Bandung as Tourist Destination : ……………………………………………………………………………………………

Three tourist attractions that you like the most in Bandung : ……………………………………………………………………………………….

………………………………………………………………………………………………………………………………………………………………………………………

ACCESS, INTERNET, SOCIAL AND MEDIA

Local simcard mobile operator used at this time : ………………………………………………………………………………………………………..

Localtion of WiFi usage : □ hotel, □ restaurant, □ tourist attractions, □ other ……………………………………………………..

The average accumulation of time in hours of internet usage in one day during Bandung : [ ………. ] hours

The three names of social media you used often : ………………………………………………………………………………………………………..

Social media used to post updates about holidays : …………………………………………………………………………………………………….

Usage of smartphone while traveling

□ Taking photos □ Video call □ Share photos

□ Map features □ Telephone □ Online banking

□ restaurant search □ Currency converter □ Stay notified by messenger

□ Search for activities and attractions □ Reading news □ As tourist guide

□ Translator □ Social media posting

Participant Signature:
